# PPARG is dispensable for bovine embryo development up to tubular stages^†^

**DOI:** 10.1093/biolre/ioae083

**Published:** 2024-06-04

**Authors:** Alba Pérez-Gómez, Leopoldo González-Brusi, Inés Flores-Borobia, Nuria Martínez De Los Reyes, Adolfo Toledano-Díaz, Antonio López-Sebastián, Julián Santiago Moreno, Priscila Ramos-Ibeas, Pablo Bermejo-Álvarez

**Affiliations:** Animal Reproduction Department, Instituto Nacional de Investigación y Tecnología Agraria y Alimentaria, Consejo Superior de Investigaciones Científicas, Madrid, Spain; Animal Reproduction Department, Instituto Nacional de Investigación y Tecnología Agraria y Alimentaria, Consejo Superior de Investigaciones Científicas, Madrid, Spain; Animal Reproduction Department, Instituto Nacional de Investigación y Tecnología Agraria y Alimentaria, Consejo Superior de Investigaciones Científicas, Madrid, Spain; Animal Reproduction Department, Instituto Nacional de Investigación y Tecnología Agraria y Alimentaria, Consejo Superior de Investigaciones Científicas, Madrid, Spain; Animal Reproduction Department, Instituto Nacional de Investigación y Tecnología Agraria y Alimentaria, Consejo Superior de Investigaciones Científicas, Madrid, Spain; Animal Reproduction Department, Instituto Nacional de Investigación y Tecnología Agraria y Alimentaria, Consejo Superior de Investigaciones Científicas, Madrid, Spain; Animal Reproduction Department, Instituto Nacional de Investigación y Tecnología Agraria y Alimentaria, Consejo Superior de Investigaciones Científicas, Madrid, Spain; Animal Reproduction Department, Instituto Nacional de Investigación y Tecnología Agraria y Alimentaria, Consejo Superior de Investigaciones Científicas, Madrid, Spain; Animal Reproduction Department, Instituto Nacional de Investigación y Tecnología Agraria y Alimentaria, Consejo Superior de Investigaciones Científicas, Madrid, Spain

**Keywords:** Peroxisome proliferator-activated receptor gamma (PPARG), conceptus elongation, lipid signaling, uterine fluid, CRISPR, embryo, epiblast

## Abstract

Following blastocyst hatching, ungulate embryos undergo a prolonged preimplantation period termed conceptus elongation. Conceptus elongation constitutes a highly susceptible period for embryonic loss, and the embryonic requirements during this process are largely unknown, but multiple lipid compounds have been identified in the fluid nourishing the elongating conceptuses. Peroxisome proliferator-activated receptors mediate the signaling actions of prostaglandins and other lipids, and, between them, PPARG has been pointed out to play a relevant role in conceptus elongation by a functional study that depleted *PPARG* in both uterus and conceptus. The objective of this study has been to determine if embryonic PPARG is required for bovine embryo development. To that aim, we have generated bovine *PPARG* knock-out embryos in vitro using two independent gene ablation strategies and assessed their developmental ability. In vitro development to Day 8 blastocyst was unaffected by PPARG ablation, as total, inner cell mass, and trophectoderm cell numbers were similar between wild-type and knock-out D8 embryos. In vitro post-hatching development to D12 was also comparable between different genotypes, as embryo diameter, epiblast cell number, embryonic disk formation, and hypoblast migration rates were unaffected by the ablation. The development of tubular stages equivalent to E14 was assessed in vivo, following a heterologous embryo transfer experiment, observing that the development of extra-embryonic membranes and of the embryonic disk was not altered by PPARG ablation. In conclusion, PPARG ablation did not impaired bovine embryo development up to tubular stages.

## Teaser

PPARG ablation by two independent gene ablation strategies did not impair bovine embryo development to tubular stages (E14).

## Graphical Abstract

**Graphical Abstract:**
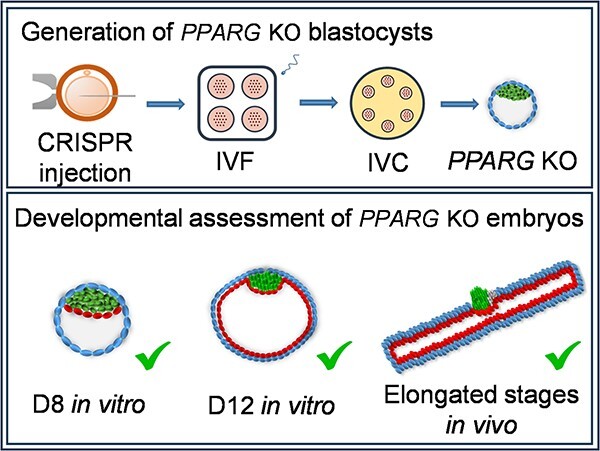

## Introduction

Preimplantation embryo development in ungulates is characterized by a prolonged post-hatching period termed conceptus elongation. Such a period is particularly relevant for the reproductive management of ungulate livestock species, as it has been deemed as the most susceptible period for embryonic loss. Focusing on cattle, different studies have estimated that embryonic losses occurring during early conceptus elongation, between blastocyst hatching and maternal recognition of pregnancy, oscillate between one third [1, 2] and up to one half of the embryos in high yielding dairy cows [3]. During this extended preimplantation period, relevant developmental landmarks occur, including the definite differentiation of three lineages, which evolve forming the embryonic disk (epiblast) and two extra-embryonic membranes (hypoblast and trophectoderm) that proliferate extensively, shaping the spherical blastocyst into an ovoid, tubular, and finally filamentous conceptus (reviewed by [4–6]).

Conceptus elongation relies on the secretions of uterine glands [7], which modulate uterine fluid (UF) composition to meet embryonic demands. Studies on UF composition have uncovered a myriad of metabolites [8–13] and proteins [14–16] potentially required for elongation. Between the different compounds present in the UF nourishing the elongating conceptus, lipids have been suggested to play relevant structural, metabolic, or signaling roles during conceptus elongation based on descriptive and experimental data. The concentrations of diverse lipids in the UF vary depending on the systemic concentrations of progesterone [11] and on the presence or absence of a conceptus [10], and the media supporting in vitro ungulate embryo development beyond blastocyst hatching contain diverse lipids absent in the conventional embryo culture media, unable to support development to post-hatching stages [17].

Peroxisome proliferator-activated receptors (PPARs) play a central role in lipid signaling. PPARs are transcription factors that dimerize with retinoid X receptors (RXRs) and regulate the transcription of target genes upon binding to PPAR-responsive elements (PPREs) [18]. Between the different PPARs, *PPARG* (PPARγ) is expressed by both the ovine embryo and endometrium [19], it co-localizes with RXR in both the trophectoderm and inner cell mass of bovine blastocysts [20], and its expression increases during elongation in bovine and ovine conceptuses [10, 19]. Further evidence for its relevant role during conceptus elongation was provided by a functional study that observed that *PPARG* knock-down induced by intrauterine infusion of morpholino antisense oligonucleotides (MAO) in ewes severely impaired conceptus elongation, leading to a reduction in prostaglandins and interferon Tau content in the UF and altered endometrium transcription [21]. However, as this approach induces *PPARG* down-regulation in both uterus and conceptus, the specific role of PPARG in the conceptus remains unexplored. The aim of this study has been to test the hypothesis of embryonic PPARG being required for early conceptus elongation by conducting two loss-of-function approaches involving CRISPR-mediated ablation in bovine embryos.

## Material and methods

### Gene ablation strategies and generation of CRISPR components

The ablation of the PPARG gene was achieved by two independent gene editing strategies. The first relies on the generation of randomly generated frame-disrupting insertion/deletion (indels) following non-homologous end-joining repair (NHEJ) of the double-strand breaks (DSB) generated by the conventional CRISPR/Cas9 system. NHEJ is an error-prone DSB-repair mechanism that introduces random indels at the repaired locus. If the randomly generated indel is not a multiple of three, it disrupts the open reading frame (ORF) of the gene, leading to a truncated protein (Supplementary Figure S1). For this strategy, a single guide RNA (sgRNA) targeting the first exon of the gene was designed to truncate the PPARG protein at the fourth amino acid (Table 1). sgRNA was synthesized and purified using the Guide-it sgRNA In Vitro Transcription kit (Takara) using a primer containing the T7 promoter and the target sequence (Table 1). Capped polyadenylated Cas9-encoding messenger RNA (mRNA) was produced by in vitro transcription using mMESSAGE mMACHINE T7 ULTRA kit (Life Technologies), and the plasmid pMJ920 (Addgene 42234) linearized with BbsI. Messenger RNA was purified using the MEGAClear kit (Life Technologies).

**Table 1:** Details of primers used

ID	Sequence (5′➔3′)	Use
T7 Guide-it Cas9	CCTCTAATACGACTCACTATAGG*AGGGTTTCACCCATCACAGC*GTTTAAGAGCTATGC	To produce gdRNA against *PPARG* for Cas9. Target sequence is underlined
GenoIl F	*TGTGAGGTCCTTGCAGACAC*AACCAATCACGTAGGCTCGG	To genotype Cas9 target in Day 12 and elongated conceptuses by miSeq. Illumina overhangs are underlined
GenoIl R	*CTGTGACAAGCTGTTGCGTC*TGATAAGGCACCTGTCAGCC	
T7 Guide-it BE3	CCTCTAATACGACTCACTATAGG*TTTCAGAAGTGCCTTGCTGT*GTTTAAGAGCTATGC	To produce gdRNA against *PPARG* for BE3. Target sequence is underlined
Geno F	TCTCCCTCGCCCATATTCCT	To genotype BE3 target in Day 8 and elongated conceptuses by Sanger
Geno R	TCGTGGCAAATGCCCAACTA	

A second gene ablation strategy relies on the introduction of a stop codon at the *PPARG* sequence by the cytosine base editor BE3 [22]. Instead of generating a DSB that will result in randomly generated indels, CBE converts cytosine to thymidine, a conversion that can generate a stop codon and thereby disrupt protein formation. For this strategy, a sgRNA was designed in the fourth exon of the gene to convert a CAG codon encoding for glutamine into a TAG stop codon and truncate the protein to 196 amino acids (39% of length) (Supplementary Figure S1). The truncated protein is not functional, as it lacks critical domains, including one essential for interacting with its constitutive coactivator, FAM120B [23], the nuclear receptor ligand binding domain, and the nine amino acid TAD motif required to interact with the transcriptional machinery factors [24] (Supplementary Figure S2). This strategy facilitates genotyping and provides an independent validation of the results obtained by the first strategy. The gdRNA was produced as described for the first strategy using a specific primer for the target sequence (Table 1). Capped polyadenylated BE3-encoding mRNA was produced by in vitro transcription using the mMESSAGE mMACHINE T7 ULTRA kit (Life Technologies), and the plasmid pCMV-BE3 (Addgene 73021) linearized with BbsI. Messenger RNA was purified using the MEGAClear kit (Life Technologies).

### In vitro production and development of bovine gene edited embryos

Bovine gene edited embryos were produced in vitro following methods previously optimized by our group [25]. Briefly, immature cumulus-oocyte complexes (COCs) were obtained by aspirating 2–8 mm follicles from bovine ovaries collected at local slaughterhouses. COCs were selected based on conventional morphological criteria and matured for 24 h in groups of 50 in four-well dishes containing TCM-199 medium supplemented with 10% (v/v) fetal calf serum (FCS) and 10 ng/mL epidermal growth factor at 38.5°C and 5% CO_2_ in air with a humidified atmosphere. Matured oocytes were denuded by vortexing for 3 min in a 300 μg/mL hyaluronidase solution and randomly divided into two groups for microinjection, one serving as microinjection control and composed only by wild-type (WT) embryos, and the other edition group microinjected with all CRISPR components (described below) and partially composed by knock-out (KO) embryos (i.e. containing non-edited, edited non-KO, and KO embryos, as explained below). For the first gene ablation strategy, the edition group (C+G) was microinjected with a solution of 300 ng/μL of mRNA encoding Cas9 and 100 ng/μL of gdRNA, whereas the control group (C) was microinjected with a solution of 300 ng/μL of mRNA encoding Cas9 alone. For the second gene ablation strategy, the edition group (BE+G) was microinjected with a solution of 200 ng/μL of mRNA encoding BE3 and 100 ng/μL of gdRNA, whereas the control group (BE) was microinjected with a solution of 200 ng/μL of mRNA encoding BE3 alone. Microinjection was conducted in Euroflush (IMV) medium.

Immediately following microinjection, IVF was performed with frozen–thawed spermatozoa from a single stud bull selected through a gradient of 40%–80% Bovipure (Nidacon Laboratories AB, Sweeden). Spermatozoa were diluted in TALP medium supplemented with 10 mg/mL heparin (Calbiochem) to a final concentration of 10^6^ sperm/mL and incubated with the microinjected oocytes at 38.5°C in an atmosphere of 5% CO_2_ and maximum humidity for approximately 20 h. Following IVF, presumptive zygotes were vortexed for 30 sec to remove the sperm attached to the zona pellucida and cultured in groups of ~25 in 25 μL droplets of synthetic oviduct fluid (SOF, [26]) supplemented with 5% FCS covered by mineral oil. Culture took place at 38.5°C in an atmosphere of 5% CO_2_, 5% O_2_, and 90% N_2_ with maximum humidity. Embryos were cultured up to Day 7 post-insemination for all experiments conducted. For developmental analysis to the blastocyst stage, edited embryos generated from the second gene ablation strategy (BE3) were allowed to develop in SOF medium to D8, when blastocyst rate was annotated and blastocysts were fixed by incubation in 4% paraformaldehyde (PFA) for 15 min at room temperature (RT), washed in phosphate buffered saline medium supplemented with 1% bovine serum albumin (PBS-1%BSA), and stored at 4°C until analysis.

### Embryo development beyond the expanded blastocyst stage

For developmental analysis of post-hatching stages (D12), D7 blastocysts generated from the first gene ablation strategy (regular Cas9) were transferred to a post-hatching culture system [27]. Under this system, embryos achieve critical developmental landmarks unreachable under conventional (SOF) culture: the complete differentiation and proliferation of the hypoblast to form an inner layer underlinning the trophectoderm and the development of an early embryonic disk. In terms of developmental progression and transcription, D12 in vitro conceptuses are roughly equivalent to E10 in vivo conceptuses [17]. However, although the system can be employed to detect early developmental failures (e.g. [28]), it does not completely mimic the in vivo conditions, as embryo development beyond E10 cannot be consistently achieved. Briefly, D7 blastocysts were transferred to 500 μL of N2B27 medium (1:1 Neurobasal and DMEM/F12 medium supplemented with 1X penicillin/streptomycin, 2 mM glutamine, and 1X N2 and 1X B27 supplements, Thermo Fisher Scientific) and cultured at 38.5°C in a water-saturated atmosphere of 5% CO_2_, 5% O_2_, and 90% N_2_, replacing half of the medium every second day. Embryo survival was analyzed at D12 (alive embryos were able to maintain the blastocoel, whereas dead embryos collapsed). Pictures were taken at D12 to measure embryo diameter using a stereo microscope (Zeiss Stemi 305) and Fiji software, and embryos were fixed as described above and stored at 4°C until analysis. The number of replicates and embryos analyzed is shown in Tables 2–4.

**Table 2:** Developmental rates in control and experimental groups up to the blastocyst stage

Group	*N*	Microinjected oocytes	Cleavage rate (mean ± s.e.m.)	Blastocyst rate (mean ± s.e.m)
BE	3	112	85 (76.1 ± 6.1)	27 (24.0 ± 4.7)
BE+G	3	310	205 (66.4 ± 4.1)	72 (23.4 ± 2.0)

Number of replicates (*N*) and microinjected oocytes. No significant differences were found (*t*-test; *P* > 0.05).

**Table 3:** Survival rates of D12 in vitro embryos from C and C+G groups

Group	*N*	D7 blastocysts in post-hatching culture	D7–D12 survival rates (mean ± s.e.m.)
C	4	20	18 (87.5 ± 8.0)
C+G	6	47	35 (76.0 ± 3.3)

Number of replicates (*N*), D7 blastocysts used for post-hatching analyses and surviving D12 embryos. Survival rates from D7 to D12 in control (C) and experimental (C+G) group (*t*-test, *P* > 0.05).

**Table 4:** Post-hatching development of D12 in vitro embryos

	*n*	Complete hypoblast migration (%)	Epiblast survival (%)	ED-like formation (%)
WT	18	8/18 (44.4)	13/18 (72.2)	7/18 (38.9)
Edited non-KO	19	12/19 (63.2)	10/19 (52.6)	8/19 (42.1)
KO	16	5/16 (31.3)	7/16 (43.8)	4/16 (25)

ED, embryonic disk; WT, wild-type; KO, knock-out.

No significant differences were found in any of the parameters analyzed between groups (Chi-square test *P* > 0.05).

For developmental analysis to further developmental stages not achievable by in vitro systems, D7 blastocysts generated from both gene ablation strategies (regular Cas9 and BE3) were transferred to two synchronized recipient ewes (one recipient used per strategy), following protocols approved by the INIA Animal Care Committee and Madrid Region Authorities (protocols PROEX 040/17 and PROEX 059.2-22). Estrous synchronization was achieved by the insertion of a progesterone-releasing device (CIDR-Ovis, Zoetis). 100 μg of cloprostenol (Estrumate, MSD) were administered 11 days after CIDR insertion, and 450 IU of PMSG (Sincropart PMSG, CEVA) were administered on the day of CIDR removal (12 days after insertion). The embryo transfer took place 8 days after CIDR removal. The presence of corpora lutea was assessed by abdominal laparoscopy, and the cranial part of the uterine horn ipsilateral to the corpus luteum (a single corpus luteus was observed in both sheep) was exteriorized to introduce 17 and 25 bovine blastocysts from the C+G and BE+G groups, respectively. Following embryo transfer, corpora lutea were maintained by CIDR insertion, and embryos were recovered 9 days after embryo transfer at a developmental stage equivalent to embryonic day (E)-14 by post-mortem [29] flushing of the uterine horns with Euroflush (IMV). The collected conceptuses were measured and fixed as described above.

### Developmental analysis by immunofluorescence

Fixed D8, D12 embryos, and elongated conceptuses were permeabilized by incubation in 1% Triton X-100 in PBS for 15 min at RT and subsequently incubated in 10% Fetal Bovine Serum-0.02% Tween 20 in PBS for 1 h at RT. Following permeabilization, specimens were incubated overnight at 4°C with primary antibodies to detect trophectoderm (CDX2, Biogenex MU392A-UC, 1:100 dilution), hypoblast (SOX17, R&D AF1924, 1:100 dilution), or epiblast (SOX2, Invitrogen 14-9811-80, 1:100 dilution) cells. After four washes in PBS-1% BSA, embryos were incubated with the appropriate secondary Alexa-conjugated antibodies (Donkey anti-rat IgG Alexa FluorTM 488, Donkey anti-goat IgG Alexa FluorTM 555, and Donkey anti-mouse IgG Alexa FluorTM 647; Life Technologies) and DAPI for 1 h at RT, followed by four washes in PBS-1% BSA. Finally, embryos were mounted on PBS-1% BSA microdrops made by drawing circles with a PAP pen (Kisker Biotech GmbH) on a coverslide and covered by an incubation chamber (Sigma Z359467) to prevent embryo crushing and PBS evaporation as described in [30]. Specimens were imaged with structured illumination equipment composed of a Zeiss Axio Observer microscope coupled to ApoTome.2 (Zeiss), which allows the visualization of z-sections. In D8 embryos, total CDX2+ and SOX2+ cells number were manually counted using the software ZEN (Zeiss). In D12 embryos, hypoblast migration, epiblast survival, and ED formation were assessed. Hypoblast migration was considered complete when all the inner surface of the trophectoderm was covered by SOX17+ cells. Epiblast survival was identified by the presence of SOX2+ cells in the embryo, and ED-like formation was identified by the presence of a compact structure containing SOX2+ cells. Hypoblast migration and epiblast survival were also analyzed in elongated conceptuses recovered following embryo transfer.

### Embryo genotyping

Following image acquisition, the D8 and D12 embryos were analyzed from the edition group, and a fragment of each elongated conceptus collected was placed at the bottom of a 0.2 mL PCR tube and stored at −20°C. D8 and D12 embryos and the conceptus fragments were digested by adding 8, 10, or 15 μL, respectively, of Arcturus Picopure DNA extraction solution (Thermo Fisher Scientific) and incubating at 65°C for 1 h, followed by inactivation at 95°C for 10 min.

Embryos generated by the first gene ablation strategy (conventional Cas9, generating indels at the target site) were genotyped by deep sequencing (miSeq Illumina), as Sanger sequencing does not allow discriminating all alleles harbored by each specimen due to the mixed sequencing reaction generated by indels of different sizes [25]. For miSeq sequencing, a first PCR was performed to amplify the sequence containing the CRISPR target site, adding Illumina adaptors using the primers detailed in Table 1, and adding 3 μL of lysate in a 25 μL PCR reaction (GoTaq Flexi, Promega). PCR conditions were as follows: 96°C for 2 min; ×32 (96°C for 20 s, 60°C for 30 s, 72°C for 30 s); 72°C for 5 min; hold at 8°C. PCR products were purified using AMPure XP beads (Beckman CoulterTM) following the manufacturer’s recommendation. A second 50 μL PCR was performed with Nextera XT Index Kit v2 primers (Illumina) to add barcodes identifying each specimen, using 5 μL of each purified PCR product as a template. PCR conditions were as follows: 95°C for 3 min; ×8 (95°C for 30 s, 55°C for 30 s, 72°C for 30 s); 72°C for 5 min; hold at 8°C. PCR products were purified by AMPure XP beads, pooled into a 4 nM library, and sequenced at miSeq (Illumina). Reads (>1000/specimen) were aligned with the WT sequence using the *BWA mem* aligner (v.0.7.17, [31]), sorted and indexed with a pipeline of different tools from the *SAM tools* package (v.1.16.1, [32]), and variants were called with *freebayes* [33]. VCF files were processed with an in-house script to select only potential indels within the CRISPR probe editing region and passing the quality check filter; variants under Q30 or sequenced with a depth under 50 were discarded, and indels should be present in both 5′-3′ and 3′-5′ reads. Results were confirmed by visually checking each VCF file with the *Integrative Genomics Viewer* [34]. Raw VCF files are available at NCBI’s SRA (accession number PRJNA1115070). Following genotyping, embryos were classified as WT (containing no mutated alleles, i.e. all embryos in the control group), edited non-KO (edited embryos containing at least one non-frame disrupting indel), and KO (containing only alleles formed by indels non-multiple of three, i.e. frame-disrupting indels). Representative visualization images of *Integrative Genomics Viewer* can be found in Supplementary Figure S3. For clearer visualization purposes (Supplementary Figure S1), clonal sequencing was performed by clonal sequencing as described in [25]. Briefly, PCR products from a WT, an edited non-KO, and a KO embryo purified using the FavorPrep PCR Purification Kit (Favorgen) were ligated into the pMD20 T-vector (Takara) by Blunt/TA Ligase (NEB) and transformed into *Escherichia coli* DH5-α competent cells. Five plasmids containing the insert were Sanger sequenced, and a WT, an edited non-KO, and a KO allele were selected to be shown in Supplementary Figure S1.

Embryos generated by the second gene ablation strategy (cytosine base editor, generating C-T substitution, but not indels) were genotyped by conventional Sanger sequencing, as in the absence of indels, alleles are clearly identifiable in the Sanger chromatogram (Supplementary Figure S1). A PCR was performed to amplify the sequence containing BE3 target site using the primers detailed in Table 1 and adding 3 μL of lysate in a 25 μL PCR reaction (GoTaq Flexi, Promega). PCR conditions were as follows: 96°C for 2 min; ×35 (96°C for 20 s, 60°C for 30 s, 72°C for 30 s); 72°C for 5 min; hold at 8°C. PCR products were purified by the FavorPrep PCR purification kit (Favorgen) and Sanger sequenced. Embryos were classified as WT (containing no mutated alleles, i.e. all embryos in the control group), edited non-KO (edited heterozygous embryos containing at least one allele not harboring the stop codon), and KO (harboring only alleles containing the stop codon).

### Statistical analyses

Data were analyzed using the GraphPad Prism (GraphPad Software, San Diego, CA, USA) and SigmaStat (Systat Software, San Jose, CA, USA) packages. Chi-square test was used to analyze the differences in the rates of complete hypoblast migration, epiblast survival, and ED-like formation between groups. Differences in developmental rates to blastocyst and D12 survival rates were analyzed by *t*-test, and embryo diameter and cell numbers between the different genotypes were analyzed by one-way ANOVA. When the normality test failed, statistical differences were analyzed by a non-parametric *t*-test (Mann–Whitney) or one-way ANOVA (Kruskal–Wallis test).

## Results

**Figure 1:**
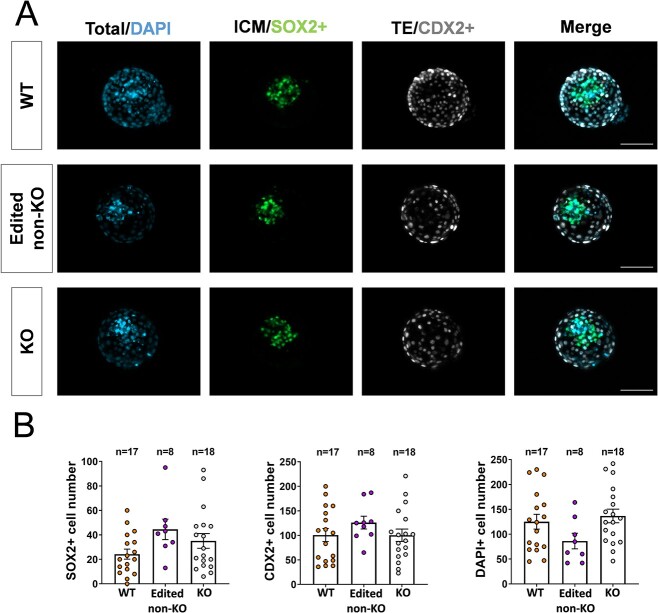
Developmental analysis up to Day 8 blastocysts. (A) Representative pictures of wild-type (WT), edited non-knock-out (non-KO), and knock-out (KO) blastocysts after IHC for SOX2 (ICM), CDX2 (TE), and DAPI (total cells). (B) Graphical representation of SOX2+, CDX2+, and total cell numbers in WT, edited non-KO, and KO blastocysts. No significant differences were found between groups (one-way ANOVA, *P* > 0.05). Scale bar: 100 μm.

PPARG ablation did not impair embryo development to the blastocyst stage. Developmental rates up to the blastocyst stage were comparable between the control group (BE, containing only WT embryos) and the edition group (BE+G, partially composed of KO embryos) (Table 2). Genotyping of 26 D8 blastocysts from the edition group revealed a 100% edition efficiency (26/26 embryos edited), and 18/26 (69%) were identified as KO (i.e. harbored only alleles containing the intended stop codon). Immunohistochemistry (IHC) analysis of the two lineages present at the blastocyst stage (inner cell mass, ICM, and trophectoderm, TE) revealed no impairment in the cell proliferation and differentiation processes, as similar ICM (SOX2+), TE (CDX2+), and total (DAPI) cell numbers were observed in all groups (Figure 1).

**Figure 2:**
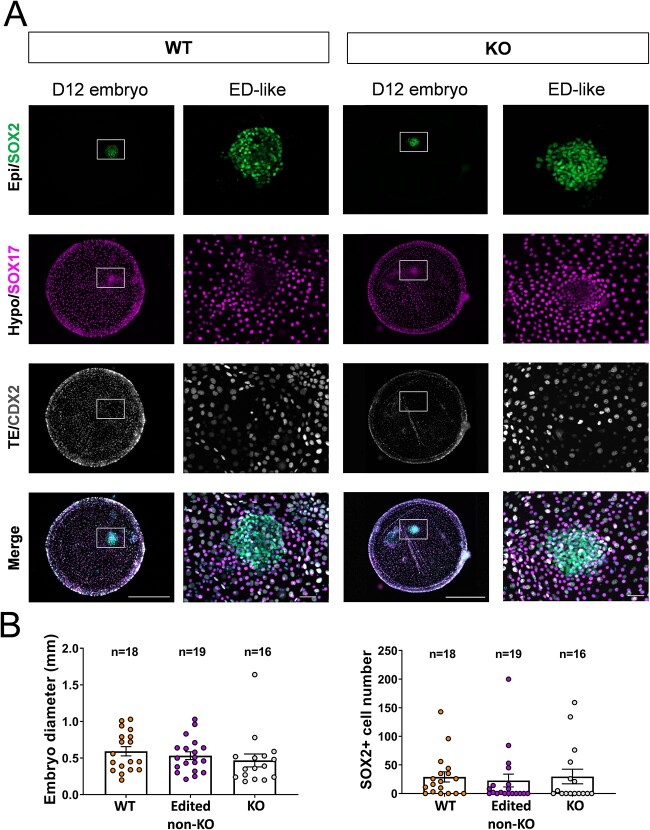
Developmental analysis up to Day 12 (post-hatching in vitro)*.* (A) Representative pictures of wild-type (WT) and knock-out (KO) D12 embryos after IHC for SOX2 (epiblast), SOX17 (hypoblast), CDX2 (TE), and DAPI (total cells). Amplifications of embryonic disks of each embryo are shown on their right. (B) Graphical representation of embryo diameter and SOX2+ cell number in WT, edited non-KO, and KO embryos. No significant differences were found between groups (one-way ANOVA, *P* > 0.05). Scale bar 500 μm for D12 embryos; 50 μm for ED-like structures.

The development to post-hatching stages in vitro was not affected by PPARG ablation. Survival rates from D7 to D12 were comparable between the control (C) and edition (C+G) groups of the conventional Cas9 gene ablation strategy (Table 3). Embryo genotyping of 35 D12 embryos revealed a 100% edition efficiency, and 16/35 (46%) were identified as KO (i.e. harbored only frame-disrupting alleles). Embryo morphology was overtly normal in KO embryos, and no differences were observed in embryo diameter compared to edited non-KO or WT embryos (Figure 2). IHC analysis showed no significant differences in hypoblast migration, epiblast survival, embryonic disk formation rates, or the number of epiblast cells between the three genotypes (Table 4, Figure 2).

**Figure 3:**
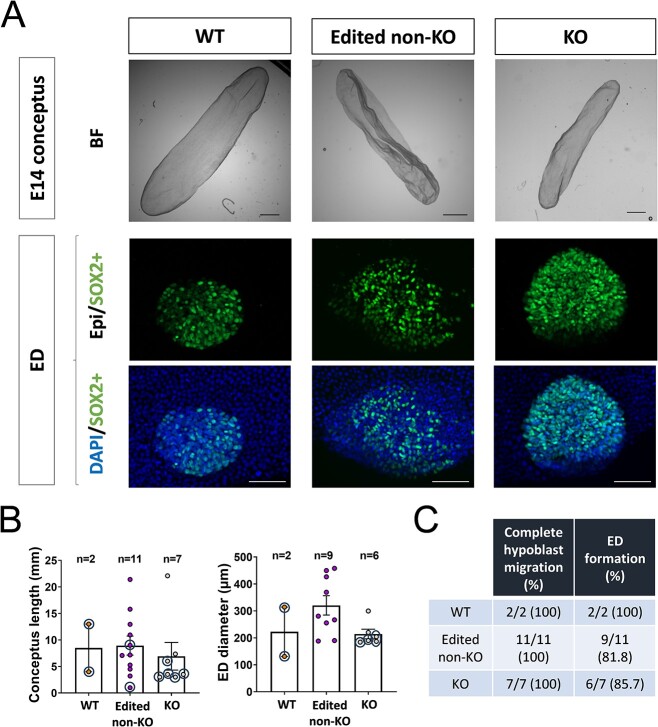
Developmental evaluation up to elongated stages in vivo*.* (A) Representative pictures in bright field (BF) of wild-type (WT), edited non knock-out (non-KO), and knock-out (KO) tubular conceptus recovered from a recipient sheep. Representative embryonic disk (ED) of each group subjected to IHC for SOX2 (epiblast) and DAPI (total cells). (B) Graphical representation of conceptus length and ED diameter (one-way ANOVA *P* > 0.05). Individual data points surrounded represent conceptuses derived from an independent replicate where base editors were used. (C) Complete hypoblast migration and ED formation rates in each group (Chi-square *P* > 0.05). Scale bar: 1 mm for conceptuses; 100 μm for EDs.

To determine if PPARG was required to attain further stages of development, edited bovine embryos generated from both gene ablation strategies were incubated in the uteri of recipient ewes to attain a developmental stage equivalent to E14. From the Cas9 strategy (C + G) group, 11 conceptuses were recovered from 17 D7 blastocysts transferred. All recovered conceptuses were edited, and two were KO (~18%). From the base editor strategy (BE+G group), 9 conceptuses were recovered from 25 D7 blastocysts transferred. Genotyping of BE+G conceptuses revealed that two were WT and seven edited (~78% edition rate), of which five were KO (56%). In both gene ablation strategies, KO embryos developed to the tubular stage and were morphologically indistinguishable from WT or edited non-KO conceptuses, showing a conceptus length within the expected range and similar to that observed for WT and edited non-KO conceptuses (Fig. 3). Embryonic disk was developed by all but one of the KO conceptuses (6/7) and embryonic disk diameter was within the expected range and similar to that observed for WT and edited non-KO conceptuses. Complete hypoblast migration inside the trophectoderm was observed for all conceptuses recovered, irrespective of their genotype.

## Discussion

Current knowledge on the embryonic requirements during conceptus elongation is limited, hampering the understanding of the frequent embryonic losses occurring during this process in cattle and other ungulates [1–3]. The presence of a myriad of lipid compounds in the UF that meet all nutritional and signaling embryonic requirements during elongation [10, 11] suggests that they may play essential roles during this developmental period. Besides, certain metabolic conditions known to reduce cattle fertility, such as the negative energy balance experienced in high-yielding dairy cows following parturition [35], are associated to altered lipidome profiles, providing a plausible link between altered systemic lipidomes and embryo development [36, 37]. PPARG constitutes a clear candidate to mediate lipid signaling in the conceptus, as it is expressed by the conceptus [10, 19], its natural ligands include unsaturated fatty acids and prostaglandins present in the UF [38–41], and its induced downregulation in both embryos and uterus impairs conceptus elongation [21]. In this context, the normal development of embryos lacking PPARG to tubular stages observed herein indicates that, instead of a direct effect upon the embryo, *PPARG* downregulation by uterine injection of MAOs [21] prevented conceptus elongation indirectly, probably by promoting changes in the UF induced by *PPARG* downregulation in endometrial cells. Given the major role of PPARG in the regulation of uterine prostaglandin secretion in ruminants [10, 42], a *PPARG*-depleted endometrium is expected to fail to secrete prostaglandins. In agreement with this hypothesis, the intrauterine infusion of an inhibitor of the main prostaglandin synthase (PTGS2) [43] resulted in a similar developmental failure as that observed following *PPARG* inhibition [21].

While *PPARG* is expressed during the entire preimplantation development [10, 19, 20], a relevant function before conceptus elongation was not observed in this study. The dispensable role of PPARG for embryo development to the blastocyst stage was expected as 1) bovine embryos develop normally to the blastocyst stage in media containing no PPARG ligand (such as SOF not supplemented with serum [26]) and 2) *PPARG* expression increases as conceptus development progresses in cattle [10, 19] and other farm ungulates, including pigs [44]. In contrast to pre-hatching development, early post-hatching development (i.e. the beginning of elongation) requires a medium containing potential PPARG ligands, such as unsaturated linoleic and lipoic acids [17]. The requirement for such compounds could be compatible with the role of PPARG during a developmental period characterized by the formation of an early embryonic disk and hypoblast migration. However, the in vitro development to those stages (D12) was not affected by the ablation, and in vivo development to further stages (E14, tubular conceptuses displaying a pre-gastrulating embryonic disk and a larger development of extra-embryonic membranes) did not require either PPARG, despite the presence of multiple potential PPARG ligands in the UF, including prostaglandins [40, 41]. The number of E14 conceptuses analyzed in this study is limited, and thereby subtle differences between KO and WT conceptuses may not have been detected, but the observation of PPARG KO elongated conceptuses showing embryonic disks overly similar to those observed in WT conceptuses clearly proves that PPARG is not required for embryo development in E14.

Although PPARG was proven to be dispensable for embryo development to E14, other PPAR family members may be required to mediate lipid signaling or regulate fatty acid metabolism during early conceptus elongation. For instance, similar to PPARG, PPARD is also expressed by nearly all trophoblastic cells of the ovine conceptus, in contrast to the scarce expression of PPARA [19], and its DNA binding activity increases during pig conceptus elongation [44]. However, in contrast to *PPARG*, MAOs-mediated downregulation of *PPARD* did not impair conceptus elongation, suggesting that PPARD may be also dispensable for elongation. Given that MAO does not completely abrogate PPARD production and that PPARG and PPARD may play redundant roles, a complex double-KO approach would be required to unequivocally solve that question.

The dispensable role of PPARG on embryo development up to the stage triggering maternal recognition of pregnancy [45] does not rule out a potentially essential role in further stages of development. Such a potential role in later developmental events, particularly on placentation processes, is supported by the known PPARG roles in non-ungulate mammals. *Pparg* KO mouse embryos are able to implant, but placental development is impaired, being poorly differentiated and showing vascular defects leading to embryonic death by E10 [46], roughly corresponding to early post-implantation stages in bovine development (reviewed by [47]). Similarly, studies in trophoblast cell lines also suggest a role for PPARG in trophoblast differentiation and invasion in mink [48] and fatty acid transport and storage in humans [49].

In conclusion, PPARG ablation did not impair bovine embryo development up to tubular stages, suggesting that the lipid signaling is either not required up to E14 or is mediated by a PPARG-independent pathway.

## Supplementary Material

New_Microsoft_Word_Document_ioae083

## Data Availability

The data underlying this article will be shared on reasonable request to the corresponding author.
