# Supplementary material for: PPARG is dispensable for bovine embryo development up to tubular stages
Source: Biol Reprod. 2024 Jun 4;111(3):557–66. doi: 10.1093/biolre/ioae083 (PMC11402522; doi:10.1093/biolre/ioae083)
Supplement: New_Microsoft_Word_Document_ioae083 [file new_microsoft_word_document_ioae083.docx]

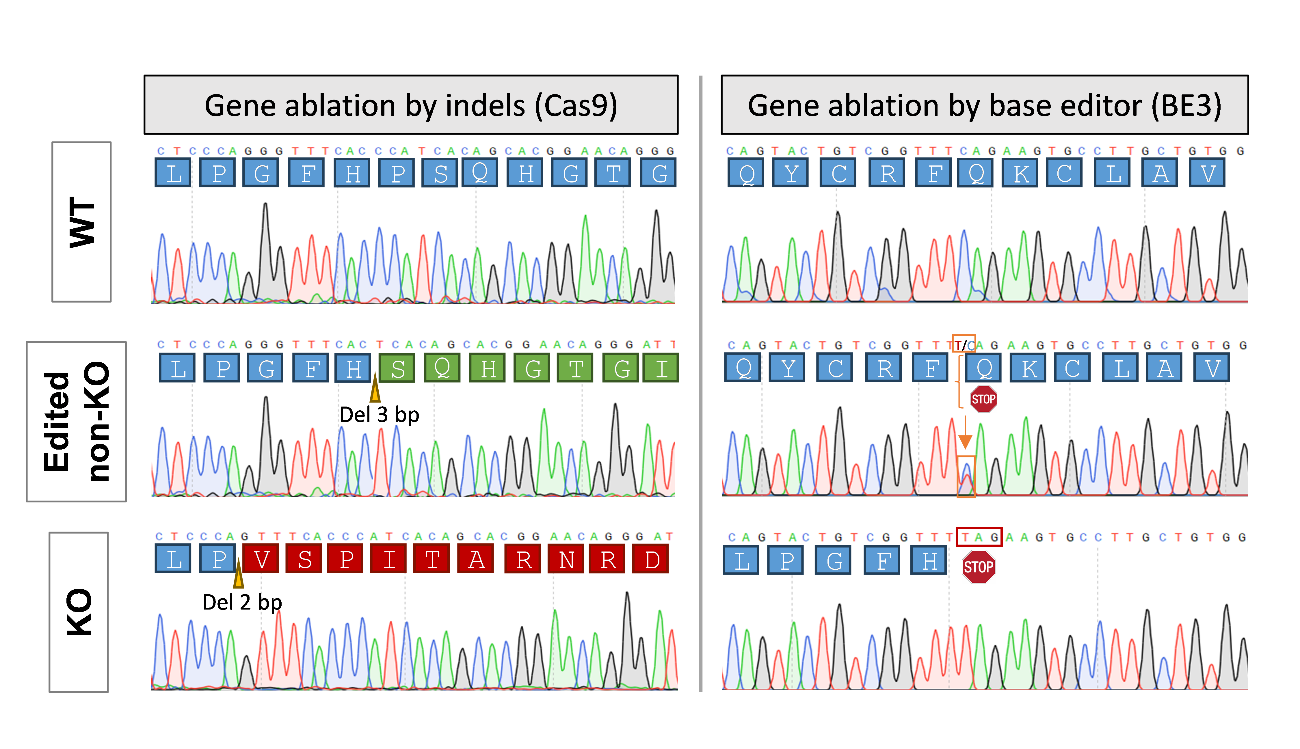


**Figure S1. Sanger sequencing chromatograms of wild-type (WT), edited non-knock-out (non-KO) and knock-out (KO) alleles/embryos following two gene ablation strategies.** Images on the left show Sanger sequencing chromatograms of alleles generated from the first strategy (conventional Cas9, generating indels at the target site). Alleles were individualized following clonal sequencing as described in [25]. Edited non-KO alleles are composed by indels multiple of three (3 bp deletion in the example) that do not disrupt the open reading frame (ORF). In contrast, in the KO allele shown in the lowest row, a 2 bp deletion alters the ORF, impeding protein translation. KO embryos are composed only by KO alleles. Images on the right show Sanger sequencing reactions of PCR products amplified from embryos generated from the second strategy (cytosine base editor, generating a stop codon at the target site). On the edited non-KO row, the PCR product of a heterozygous (WT/KO) embryo show two nucleotides on the same position, indicating the presence of two alleles (marked with orange arrow: C in the WT allele and the intended T generating a TAG stop codon in the KO allele). The lower row depicts the sequencing reaction of a KO embryo where a C-T conversion has created a stop codon in both alleles.


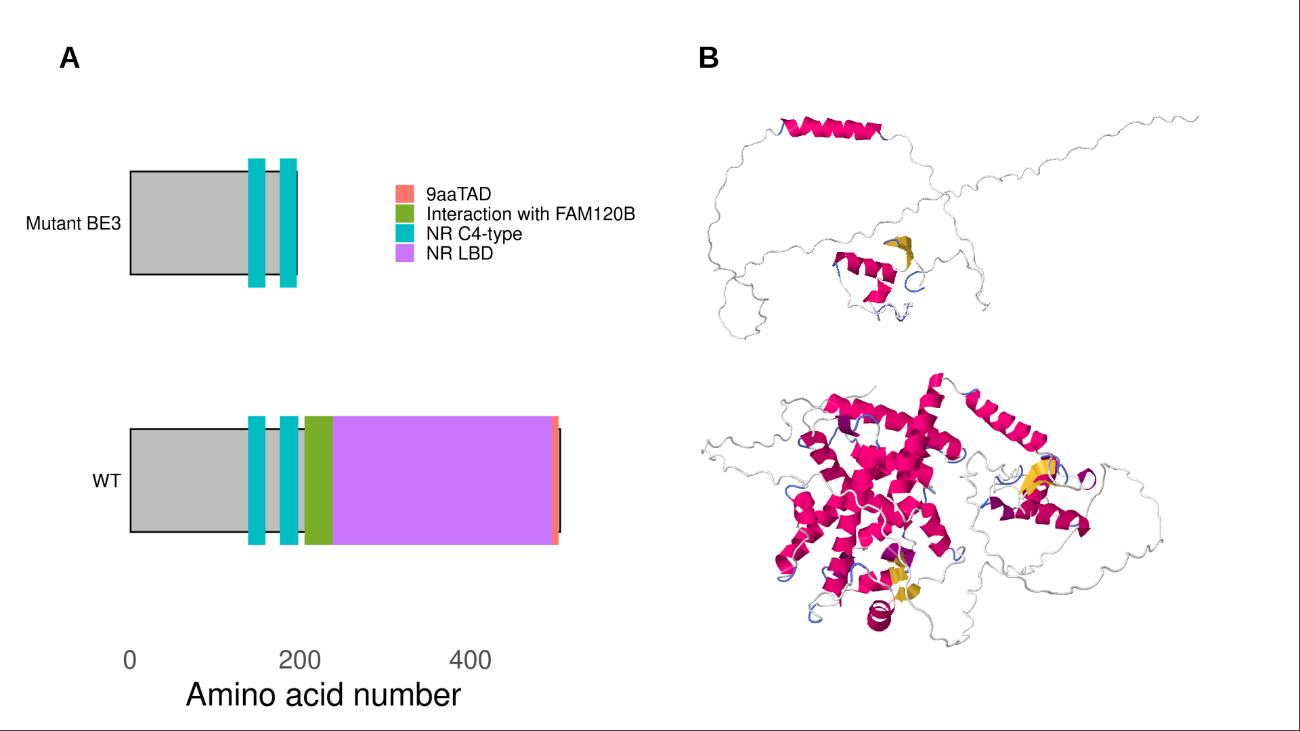


**Figure S2. *In silico* analysis of the truncated protein generated by the second gene ablation strategy (Mutant BE3).** A) Scheme depicting the truncated protein formed by the first 196 (upper image) compared with the WT protein (lower image). The truncated protein lacks critical domains for functionality, including the interacting domain with FAM120B, the nuclear receptor ligand binding domain (NR LBD), and the 9 amino acid TAD motif required to interact with the transcriptional machinery factors. B) Alpha Fold (v 2.3.2, [50]) predicted structures of the truncated (upper image) and WT (lower image) proteins.

**
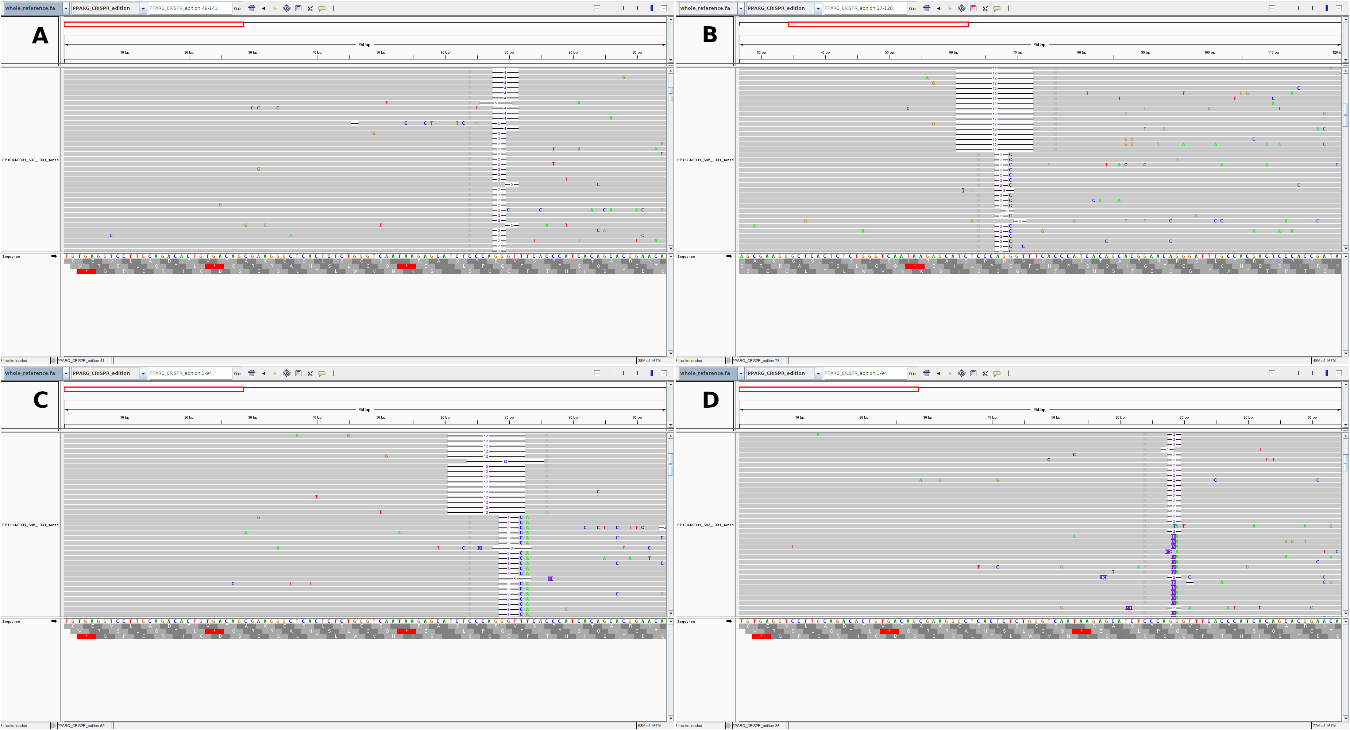
**

**Figure S3. Representative screenshots of the visualization of miSeq data under Integrative Genomics Viewer.** A) KO embryo harbouring two KO alleles formed by the deletion of 4 and 2 bp. B) Edited non-KO embryo harbouring one KO allele formed by a 2 bp deletion and another non-KO edited allele formed by a 12 bp in-frame deletion. C) Edited non-KO embryo harbouring two non-KO edited alleles formed by two in-frame deletions of 12 and 3 bp. D) KO embryo harbouring two KO alleles formed by a deletion of 2 bp and an insertion of 8 bp.
